# Correlation Between Semen Quality and Bilateral Scrotal Skin Temperature Shown in the Chronological Results of Two Patients With Left-Sided Varicocele

**DOI:** 10.7759/cureus.79047

**Published:** 2025-02-15

**Authors:** Yohei Kaizuka, Nobuyuki Kondoh, Shinpei Yoshioka, Seiji Nagasawa, Yoshikazu Togo, Akihiro Kanematsu, Shingo Yamamoto

**Affiliations:** 1 Department of Urology, Kawanishi City Medical Center, Kawanishi, JPN; 2 Department of Urology, Hyogo Medical University, Nishinomiya, JPN; 3 Department of Urology, Hyogo College of Medicine, Nishinomiya, JPN

**Keywords:** chronological measurement, heat stress, intratesticular temperature, scrotal skin temperature, thermal imaging camera, varicocele

## Abstract

Scrotal skin temperature (SST) reflects intratesticular temperature, the elevation of which has adverse effects on bilateral spermatogenesis. The present study evaluated the effects of bilateral heat stress on the semen quality of two patients with a left-sided varicocele using chronological SST measurements. Scrotal skin temperature in two patients with a left-sided varicocele was determined using a thermal imaging camera (FLIR C2, FLIR Systems, Wilsonville, OR, USA). Case 1 was a single man seeking preconception care and diagnosed with a painful Grade 2 varicocele. The value for left side SST-right side SST (ΔSST) decreased from 3.2°C to -1.2°C along with the exacerbation of the varicocele from Grade 2 to 3. A varicocelectomy was performed because of the worsening of semen quality. Case 2 was an infertile man with a painful Grade 1 varicocele. The initial ΔSST of 2.4°C was temporarily reduced to 0.6°C, followed by recovery to 2.6°C along with a clear improvement in spermatogram results. To the best of our knowledge, the present chronological findings are the first to show that bilateral elevation of intratesticular temperature in a patient with a left-sided varicocele is substantially related to the deterioration of spermatogenesis.

## Introduction

The pampiniform plexus, a network of many small veins found in the human male spermatic cord around the testes, acts as the counter-current heat exchange system [1] for cooling blood in adjacent arteries. So, its malfunction could lead to local hyperthermia. Abnormal tortuosity and dilatation of the testicular veins, termed varicocele, is observed in 35% of all males with primary infertility and in up to 81% of those with secondary infertility [2], while surgical varicocele treatment has been performed for more than 40 years for affected patients [3,4]. Spermatogenesis is a temperature-sensitive process in which hyperthermia is a deleterious condition. Nearly half a century ago, a study found a significantly higher level of intrascrotal temperature in varicocele patients as compared to a control group, with a mean difference of 0.6°C to 0.8°C [5]. Since that time, testicular hyperthermia has been one of the most studied pathophysiological factors related to varicocele. Another study found that testicular temperature was bilaterally elevated in infertile males with either unilateral or bilateral varicoceles and then reduced bilaterally following a varicocelectomy [6]. Furthermore, several studies have examined and reported the detrimental effects of a left-sided (unilateral) varicocele on the bilateral testes, including a study of testicular biopsy results for patients with a unilateral varicocele that revealed bilateral adverse effects on spermatogenesis shown in histological examinations [7].

In this context, we speculated that the detrimental effect of a left-sided varicocele on spermatogenesis is induced by the elevation of testicular temperature on the ipsilateral (left) side and then becomes bilateral, involving the contralateral side over time. To the best of our knowledge, a long-term survey for monitoring testicular temperature on each side in patients with a left-sided varicocele was performed for the first time in the world. Since intratesticular temperature is strongly correlated with scrotal skin temperature (SST) [8,9], non-invasive measurements of SST were performed with the use of an infrared thermometer.

## Case presentation

Patients

Two patients, each with a left-sided varicocele, provided written informed consent to participate in this study. Varicoceles were graded according to criteria reported by Dubin and Amelar [10] (Grade 0/subclinical: impalpable varicocele but detected by imaging, usually ultrasonography; Grade 1: barely palpable with Valsalva maneuver; Grade 2: palpable without Valsalva maneuver, but not visible; Grade 3: easily visible). Case 1 was a 30-year-old single male who had a complaint of left scrotal pain and desired a preconception check. A Grade 2 varicocele was detected at the initial visit. Case 2 was a 40-year-old married male who hoped to father a child. This patient had a Grade 1 varicocele that was painful. According to the treatment strategy of our department, a varicocelectomy was recommended to both patients, though neither desired to soon undergo surgery and agreed to participate in this study during the follow-up period.

Measurement of SST

**Figure 1:**
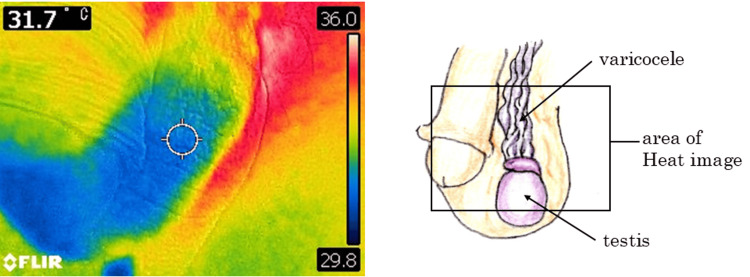
Digital display of thermal imaging camera at time of measurement Left half: Heat image; the measurement value is digitally displayed on the left upper side of the screen; Right half: The nature of varicocele is the dilatation of testicular veins composed of pampiniform plexus The image on the right has been created by the authors.

Prior to the examination, each patient was asked to undress the lower part of the body for cooling and remain in a seated position for five minutes, and then a standing position for another five minutes in a room with a constant temperature of 20~22 ℃. Subsequently, SST was measured using a thermal imaging camera (FLIR C2, FLIR Systems, Wilsonville, OR, USA) with the following technical specifications: a resolution of 76,800 pixels, a temperature range of -10°C to 150°C with an accuracy of ±2°C or 2%, and a spectral range of 7.5 µm to 14 µm. The camera was held in a single examiner’s hand so as to position it perpendicular to the scrotal skin surface and more than 0.15 m away from the body for focusing on the measurement site, according to the instruction manual. The same examiner pressed the switch to capture the thermographic image and obtained the measurement value digitally displayed on the left upper side of the screen (Figure 1).

Previously, Takada et al. reported that SST for the upper part of the scrotum is most reflective of a temperature change induced by the transition from a supine to a standing position [11]; thus, the SST was also determined at one point in the upper part of the scrotum, except for the case that displayed value could be influenced by a technical problem or machine instability. Using SST data obtained from both sides, the following calculation was performed: left side SST-right side SST (ΔSST). The sum was determined as ΔSST (°C).

Semen analysis

Semen analysis was performed according to the WHO guidelines [12]. Samples were obtained by masturbation after at least three days of abstinence. For the present study, total motile sperm count (TMSC) was used, as it is considered to be the most comprehensive and reliable seminal parameter for the evaluation of spermatogenesis.

Case 1

**Figure 2:**
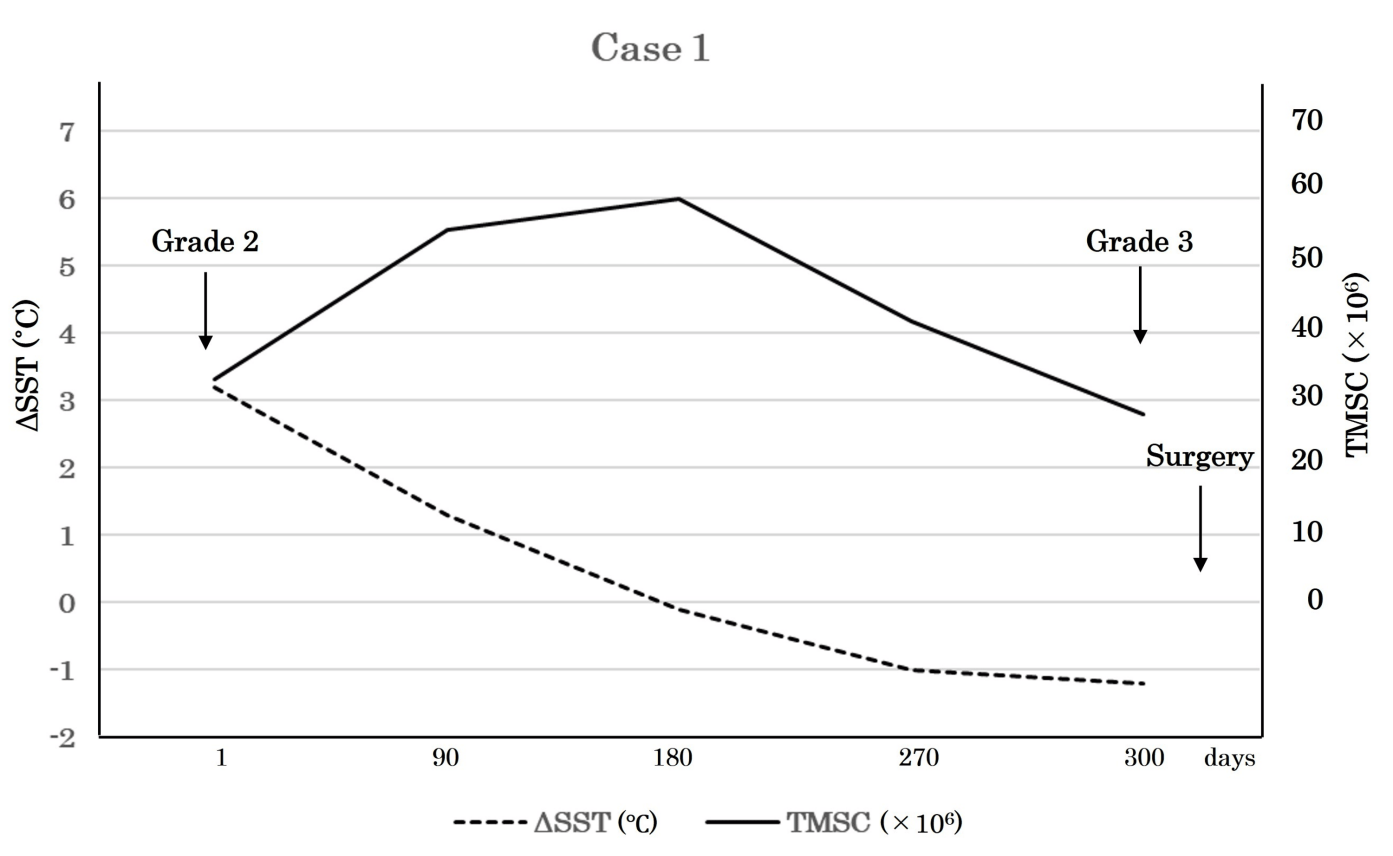
Clinical course of Case 1 After Day 180, right side SST continuously increased along with the decrease of ΔSST and final worsening of TMSC, which could be induced by the progress of varicocele grade SST: scrotal skin temperature; TMSC: total motile sperm count; ΔSST: left side SST–right side SST

On the first measurement day (Day 1), the left side SST was 31°C and the right side SST was 27.8°C, for a ΔSST value of 3.2°C. Thereafter, ΔSST fluctuated from -0.1°C (Day 180) to -1.2°C (Day 300). Exacerbation of the varicocele from Grade 2 to Grade 3 was observed in the examination conducted on Day 300. The TMSC improved from 33.1 to 60 million but then dropped again to 27.9 million. As a result, it was decided to perform surgery (Table 1, Figure 2).

**Table 1:** SST and TMSC time courses for Case 1 SST: scrotal skin temperature; TMSC: total motile sperm count; ΔSST: left side SST–right side SST Day 1 is the first SST measurement day

Day	Left side SST (˚C)	Right side SST (˚C)	ΔSST (˚C)	TMSC (×10^6^)
1	31.0	27.8	3.2	33.1
90	32.1	30.8	1.3	55.4
180	29.7	29.8	-0.1	60.0
270	31.0	32.0	-1.0	41.9
300	31.6	32.8	-1.2	27.9

Case 2

**Figure 3:**
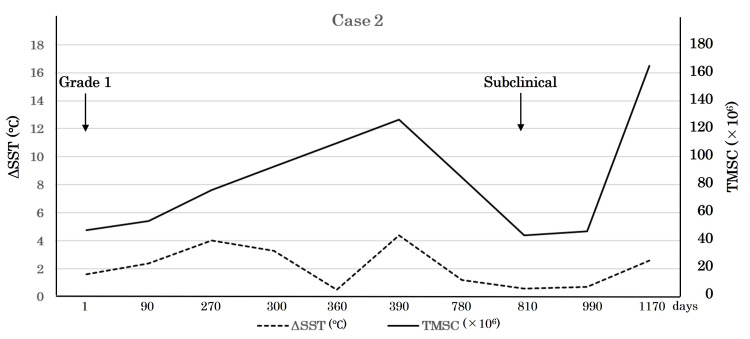
Clinical course of Case 2 There was no tendency of the right-side SST to increase, which was revealed in Case 1. The increases of ΔSST seen coupled with the improvement of TMSC, especially on Day 390 and Day 1,170, could explain the bilaterality of varicocele pathology. SST: scrotal skin temperature; TMSC: total motile sperm count; ΔSST: left side SST–right side SST

Examinations on Day 1 showed left side SST of 33.1°C and right side SST of 31.5°C, for a ΔSST value of 1.6°C. Thereafter, ΔSST generally diminished, with a peak of 4.4°C on Day 390 and then down to 0.6°C on Day 810. However, after the varicocele was downgraded from Grade 1 to a subclinical level, ΔSST increased to 2.6°C along with a decrease in right side SST. The TMSC fluctuated from 54 to 126.7 million and then down to 43.9 million, though it was clearly improved to 164.8 million on Day 1,170 along with an increase in ΔSST (Table 2, Figure 3).

**Table 2:** SST and TMSC time courses for Case 2 SST: scrotal skin temperature; TMSC: total motile sperm count; ΔSST: left side SST–right side SST Day 1 is the first SST measurement day.

Day	Left side SST (˚C)	Right side SST (˚C)	ΔSST (˚C)	TMSC (×10^6^)
1	33.1	31.5	1.6	47.7
90	32.1	29.7	2.4	54
270	33.7	29.7	4.0	76.3
300	32.1	28.8	3.3	93.1
360	31.2	30.7	0.5	109.9
390	32.2	27.8	4.4	126.7
780	31.4	30.2	1.2	85.3
810	33.0	32.4	0.6	43.9
990	30.7	30.0	0.7	46.5
1,170	32.9	30.3	2.6	164.8

## Discussion

The generally accepted pathophysiology of varicocele for spermatogenesis consists of three linked processes: heat stress due to hyperthermia, excess reactive oxygen species (ROS), and increased apoptosis. Transient scrotal hyperthermia (heat stress) has been shown to cause impaired spermatogenesis through excess ROS in rodents [13] and humans [14]. Furthermore, previous studies have demonstrated decreases in preoperative elevation of SST [6] and testicular oxidative stress (OS) [15,16] after undergoing a varicocelectomy.

Detrimental effects of a unilateral varicocele on the bilateral testes have been demonstrated [17,18], including histological evidence in humans [7]. Accordingly, we consider that spermatogenesis is affected and subsequent deterioration of seminal parameters occurs after the involvement of the contralateral testis as an adverse effect in unilateral varicocele cases. In other words, a clinically noted spermatogenesis disorder is a bilateral event in patients with a unilateral varicocele. Therefore, should a varicocele influence only the ipsilateral side, though difficult to detect preoperatively, a varicocelectomy might not result in a therapeutic effect, which is often noted in clinical situations.

For the present patients, SST, which reflects intratesticular temperature (ITT) and is a potent marker of heat stress, a well-known adverse effect, was examined on both sides. Related to our earlier noted speculation regarding the pathophysiology of a left-sided varicocele, elevation of SST (ITT) on an ipsilateral (left) side has been shown to be initially provoked by destruction of the counter-current heat exchange system between arteries and pampiniform veins [1]. As a result, elevated ITT propagates in the contralateral (right) testis due to close proximity, leading to elevation of bilateral SST over time. Therefore, it is considered that the destruction of counter-current heat exchange on the right side may not be necessary for the bilateral elevation of SST.

There are two relevant points highlighted by the sequential data obtained in examinations of these two left-sided varicocele patients. First, the elevation of SST on the right side may be negatively correlated with TMSC, which is independent of the degree of elevation of the left side SST. Second, a decrease in ΔSST is likely the consequence of an increase in right-side SST, not a decrease in left-side SST. Together, movement of right-side SST is likely a crucial factor for seminal quality and subsequent necessity of a varicocelectomy, indicating that a testicular spermatogenetic disorder in a unilateral varicocele patient should be recognized as a bilateral disease.

In regard to factors indicating a left-side varicocelectomy, we previously reported that a Grade 2-3 varicocele should be repaired because of a significant reduction in left testicular volume [19]. Conversely, it can be considered that the presence of a unilateral left-sided varicocele less than Grade 2 indicates that bilateral testicular function has already been affected. Actually, recovery of semen quality after repair of a rather small varicocele has been noted in some cases, which is well understood when considering that testicular varicocele formation in infertile men is a bilateral condition. A cut-off value for right-side SST as a tool for the determination of the bilaterality of adverse effects was not used; thus, further studies that include a control group will be required.

The primary limitation of this prospective study is the low number of subjects, likely because of the long observation period prior to treatment required. Although lack of data after varicocelectomy may be a second limitation, a postoperative decrease of SST was confirmed literally [6]. In this regard, we consider that the relationship between SST and semen parameters should not be specialized in the case of varicocele. Furthermore, obtained data are not indicative of statistical analysis findings, which may be another limitation.

## Conclusions

The two patients in the present study with a left-sided varicocele showed completely opposite progress in follow-up examinations. As a result, one underwent a varicocelectomy after elevation of right side SST, while the other showed improved semen quality, especially TMSC, along with a bilateral decrease of SST and varicocele downgrading. These are the first chronological findings of bilateral elevation of intratesticular temperature in patients with a left-sided varicocele, which is substantially related to spermatogenesis deterioration. 
